# Clinical Lecture in the Medical Wards of the Mercy Hospital, Nov. 19th, 1855; Being the 39th Clinique of the Present Course

**Published:** 1855-12

**Authors:** N. S. Davis

**Affiliations:** Prof. of Practical and Clinical Medicine in Rush Medical College, Chicago, Ill.


ART. III.—*Clinical Lecture in the Medical Wards of the Mercy Hospital, Nov. 19th, 1855; being the 39th Clinique of the present Course.* By N. S. DAVIS, M. D., and Prof. of Practical and Clinical Medicine in Rush Medical College, Chicago, Ill.

GENTLEMEN:—I shall this morning again occupy your time on the common, but all important subject of Fevers, and their local complications. But before entering the wards to take our places by the bed-side of the sick, I wish to call your attention to a portion of the small intestine and mesentary which was taken from the dead body only two or three days since. You will remember that I showed you not long since, a portion of the rectum which illustrated the extreme changes produced in the mucous membrane of that part during protracted and severe dysentery.

During the same interview I called your attention to the mucous membrane of a portion of the ilium or small intestine, in which you saw several of the eliptical plates or patches of Payer's glands, thickened and elevated above the surface of the surrounding membrane, and presenting numerous small points or indentations, giving to the surface what has been called the *shaven beard* appearance. In the same thickened and elevated patches you saw many points looking like a deposite of gray or yellowish gray tubercular matter beneath the epithelial covering of the mucous membrane.

The only appearance of actual ulceration which you saw in that pathological specimen, consisted of one solitary ulcer, situated in a plica or fold of the mucous membrane, and was about half the size of an ordinary pea. I explained to you, that the appearances then observed were those frequently seen in the mucous membrane of the small intestines after death, from what the books technically call typhus fever. The pathological specimen which I now hold in my hands, is a section of the ilium and mesentery, from a subject dead from protracted typhoid fever with severe intestinal symptoms. The suject had also been previously afflicted with scrofulous disease of the lymphatic glands in the neck. The intestine, as you see, has been laid open to expose more fully its interior or mucous surface; and if you observe closely you will easily distinguish here a portion of the membrane, deeper color, and more elevated than the surrounding surface. Its margin is well defined ; and it constitutes one of those "eliptical plates," so minutely described by M. Louis, in a state of simple thickening and increased vascularity. It differs from the *plates* or patches of glands in the specimen I showed you a few days since, in presenting no *shaven beard* appearance or indications of a deposite under the epithelium. There are several other spots of precisely the same character in this intestine, but here is one in which the mucous membrane has been entirely destroyed leaving, as you see, a superficial ulcer as large as a half dime, with irregular and slightly elevated edges. Here is another still larger, but presenting the same characteristics; and here is a place where it is evident that two of the patches of glands have been destroyed by ulceration together with the intervening mucous surface, so that we have a continuous ulcerated surface almost equal in extent to a silver half-dollar. The edges are seen to be very irregular and in laying the intestine open the incision was carried through a part of the ulcer, separating it upon the other margin which you see here. These different portions of the interior surface of the intestine, present only different degrees of advancement of the same morbid process. The first characterized by increased vascularity, thickening, and softening of texture, is only an earlier stage of the same process which, at other points, has resulted in ulceration and complete destruction of the membrane. If you turn your attention to this adjoining portion of the mesentery, you see the glands much enlarged. Here is one which has attained the size of a hickory nut, and others present all sizes down to that of a small pea. The only outward mark of disease besides the swelling consists of this small patch of inflammatory redness over one side of the largest gland. The structure of all these glands is changed, however, being softened and in some of them entirely disorganized. This one which has been laid open, presents an interior consisting of a whitish, pultaceous, almost semi-fluid mass of matter. These morbid appearances which you have now carefully examined, are just such as usually take place, to a greater or less extent, in all protracted cases of typhoid fever, accompanied by a tympanitic abdomen and thin or liquid intestinal discharges. It is these lesions, both in the intestine and mesentery, which have been regarded by M. Louis and others of the same pathological school both in Europe and America, as the essential pathological feature of this variety of continued fever. Indeed, it has been claimed that these intestinal ulcerations, bear the same relation to the typhoid fever, that the eruptions on the skin do to the fever of variola or scarlatina. Hence it has been styled *dothinentirites* by some European Continental writers, folicular-enteritis, and entero-mesenteric Fever, by others; and simply *Enteric Fever* by Dr. Wood. Gentlemen, I cannot coincide with these exclusive views in regard to the pathology of typhoid fever.

On the contrary, I am constrained, both by observations at the bed-side and by post mortem dissections, to regard these intestinal and mesenteric lesions, in precisely the same light as the alterations in the texture of the spleen, the liver, the lower lobes of the lungs, the heart, and the brain, which are often seen accompanying fatal cases of this variety of fever.

All these changes are doubtless *consequences*, developed during the progress of the general fever, but constituting no part of its constant and necessary pathology. It is true that some are developed more frequently than others, and exert a greater influence on the ultimate result. If you now accompany me to the upper ward, I will direct your attention to two cases of well marked typhoid fever, in the advanced stage of their progress, both of which present local complications of importance but differing much from each other. Here you see a young man, native of Ireland, aged about twenty-two years, who was admitted into the hospital about ten days since, having been previously confined to his bed four or five days. I called your attention to the case and pointed out all its characteristic features soon after its admission here. You will remember that, at that time his face was suffused with redness; his expression of countenance dull; his eyes sunken; his lips dry, the upper one a little retracted and it together with the ends of the upper teeth covered with brown sordes; the mouth dry; the tongue red at the tip and edges, but its upper surface covered with a dry, brown, coat; his intellect and special senses dull; the skin generally dry, rough, and moderately hot; the pulse one hundred per minute, small and soft; the abdomen moderately full, tympanitic, and slightly tender on pressure; the discharges from the bowels were frequent, amounting to six or eight times in twenty-four hours, and accompanied by some pain in the abdomen; the matter discharged being thin and of a brown or reddish brown color, and sometimes intermixed with a little mucus. The urine was scanty and the respiration feeble. I then explained to you that the symptoms presented were plainly indicative of a severe typhoid fever with special lesions in the mucous membrane of the alimentary canal.

The latter were particularly indicated by the intestinal discharges, the tympanitic and gurgling abdomen, and the dry tongue with red edges; while the general type of fever was determined by the soft, compressible pulse; the moderately hot but very dry skin; the diffused redness of the face; the dulness of intellect and special senses; and the absence of well marked periodicity. In reference to the prognosis, I pointed out two leading sources of danger. The one consisted in the direct failure of the organic actions, dependent on that diminution of elementary *susceptibility* or irritability of all the tissues, which constitues an essential element in the true typhoid condition. The evidence of this failure of organic action and diminished susceptibility throughout all the tissues, is found in the torpor of intellect, the dulness of special senses, including the nervous sensibility generally, the slow and hesitating muscular movements, the feeble capillary circulation, the general diminution of the secretions, and in the darker color and slower coagulability of the blood when drawn from the veins and allowed to stand in an open vessel. These phenomena are all independent of any mere local lesion, and arise directly from an altered condition of the properties of both solids and fluids throughout the system. This altered condition is sometimes of itself sufficient to destroy the life of the patient. This is indeed frequently the case in the lower grades of typhoid disease, such as occurs in camps, jails, crowded emigrant ships, etc. In the ordinary typhoid fever of the country, however, these changes are generally more moderate, and seldom constitute the immediate cause of death; and still they are constant elements in this variety of fever, giving to it that tendency to *debility* or *typhoid prostration*, against which the books so uniformly caution us. It is of much practical moment to appreciate clearly these general alterations in each individual case, as on them should be founded one of the leading indications for treatment.

The other source of danger in the case before you, was attributed to the changes which were taking place in the mucous membrane of the intestines. The precise nature and tendency of these abdominal lesions, as demonstrated by the researches of Louis, Chomel, Gherard, Jackson, and others, were fully pointed out to you at the previous examination, and need not be repeated at this time. You will remember that the opinion was then expressed, that the same morbid change had commenced in the aggregated glands or eliptical plates of the mucous membrane of the ilium, and the glands in the corresponding portions of the mesentery, as you have just now examined in the pathological specimen exhibited in the room below.

The vascularity of these parts was already increased, marking the commencement of that change which results in softening and ulceration. The case then presented two clear and well defined indications for treatment. The first was to arrest the further impairment of the properties of the blood and tissues, and thereby prevent an undue or dangerous degree of general depression or prostration. The second, was to counteract the morbid change going on in the glandular structures of the mucous membrane of the intestines and mesentery.

Having pointed out as clearly as possible the indications for treatment, the next inquiry was in reference to the best means for fulfilling them. To meet the first, you will recollect, the patient was directed a regular supply of nourishment, consisting of animal broth well seasoned with salt, or sweet milk boiled with a little flour. Those articles were chosen, as you are aware, chiefly because they contain a sufficient proportion of nutritive matter in a state easily absorbed from the stomach and first part of the intestines, and leaving but little fœcal residue to pass over the morbidly sensitive and diseased surface of the ilium and colon; qualities of much importance in the intestinal variety of typhoid fever. The nourishment should be given in moderate quantities and at regular intervals, without much reference to the appetite of the patient. To still further maintain the tonicity of the tissues, and arrest the tendency to impairment of the quality of the blood, I directed for the patient, a solution of chloride of sodium, sulph. quinine, and sulph, morph., in water, in such proportion that the patient would get in each dose 15 grs. of the first, 2 grs. of the second, and ¼ of a grain of the third, every four hours. When I prescribed this at our former interview with this patient, some of you who are conversant with the prevailing doctrines concerning the treatment of typhoid fever, may have regarded it as inefficient for sustaining the forces of the system, and may have been ready to ask why the more diffusable stimulants, such as brandy or wine, or bark, were not resorted to as recommended by Dr. Stokes of Dublin, and most other modern teachers. To answer this question fully would require more time than is now at my command. If you remember what I have already stated in the lecture room concerning the condition of the blood in the fully formed typhoid disease, you will have reason to suppose that in the patient before us, that fluid is of a darker hue and its fibrine less coagulable than in health; and consequently it is less capable of producing its normal impression on the elementary properties of the tissues, by which all the functions of the system are maintained. If this is true, we should certainly select as far as possible such remedies as will arrest this change in the blood and restore its healthy qualities; for in so doing we shall most efficiently sustain all the organic processes and function.

But if there is any reliance to be placed on the experiments of Prout, Bouchardet, Sir Astley Cooper, Bernard or myself, it is certain that alcohol when introduced into the system directly lessons both the exhalation of carbonic acid and the absorption of oxygen from the lungs, renders the blood darker color, lessens the coagulability of the fibrine, and diminishes all the organic actions. Hence unless we actually prescribe on the fictitious principle of similia similibus curantur, we shall be forced to declare the whole class of alcoholic beverages contra-indicated in the class of patients before us. You may be disposed, however, to claim that *experience* has proved their efficacy and clearly demonstrated a less ratio of mortality under their use than under other modes of treatment. Let me caution you here against one source of fallacy in reference to this matter. You must keep in mind the fact that before the researches of Louis, Chomel, Gherard, Jackson, and others in relation to the intestinal lesions so common in typhoid fever, had become generally known to the profession, it was the general custom in the treatment of all continued fevers to resort to emetics, cathartics, and sometimes blood-letting.

In other words it was customary to employ an evacuant and strongly purturbing treatment, with a low diet. Of course many patients sunk early from exhaustion, and many more from protracted and extensive ulceration of the mucous membrane of the intestines. It is doubtless true that the comparisons instituted between the results of this practice and that from a moderate use of alcoholic stimulants coupled with sufficient nourishment, show a balance in favor of the latter. This, however, only proves that the stimulants were *less injurious* than the evacuant and purturbing methods of treatment, but leaves the question in regard to their positive value entirely unsettled. There are agents within our reach, which, when introduced into the blood, directly increased its arterial hue, add to its capacity for absorbing oxygen, lessen the tendency to alteration or degeneration of its corpuscles, and thereby render it more capable of supporting all the properties and functions of the system. These are the agents clearly indicated in the case before us; and among them I am satisfied from much and careful observation at the bed side, that there is not one among them more efficient and valuable than the chloride of sodium or common salt. Hence it was that we selected it with small doses of quinine to counteract those changes in the blood and tissues which were going on in the patient now lying before you. To fulfill the second indication, namely, the removal of the local intestinal lesion, I directed a fluid drachm of the following emulsion to be given between each of the doses of the salt and quinine solution, viz.:

℞ Ol. Terebinth, ʒji.

Tinct. Opii., ʒji.

Pulv. G. Arabac, }

White Sugar, } aa. ʒjii.

Rub together thoroughly and add

Mint Water, ℥ji. mix.

The patient continued these remedies, with very little variation for five or six days, during which time all symptoms improved to such an extent that he was deemed convalescent.

The general fever entirely subsided, the intestinal discharges become more natural both in consistence and frequency, and his appetite returned. In this state the emulsion was discontinued, and the solution of salt and quinine continued only three times a day. For two or three days the patient appeared to be doing well, but indulging too freely in the use of food, the intestinal discharges of a thin brown, character were renewed, and you now find him again, with a tympanitic abdomen, a red tongue, a pulse one hundred per minute and small, but no febrile heat or thirst. You examine the patient carefully and you will find, all the evidences of a return of that peculiar irritation in the mucous membranes, which existed on his first admission into the Hospital, but without general febrile action. This is not an uncommon occurrence during convalescence from this variety of fever, and should impress upon your minds strongly the necessity of watching closely the diet and whole management of patients convalescent from the intestinal typhoid fever until the health is fully restored. Without this you will have frequent occurrences like this, and sometimes a rapid developement of ulceration, with an ultimately fatal result. To again allay the intestinal irritation in this patient we will restrict his diet according to the rules before mentioned and give him one of the following pills every two hours, viz.:

℞ Nitras Argenti, 8 grs.
Pulv. Opii., 20 grs. Mix and divide into twenty pills. There is another patient with typhoid fever complicated with disease within the chest, to which I intended to direct your attention, but as the clinique hour has already expired, we must defer it until to-morrow morning.

